# Complications or rather side effects? Quantification of patient satisfaction and complications after orthognathic surgery—a retrospective, cross-sectional long-term analysis

**DOI:** 10.1007/s00784-020-03664-z

**Published:** 2020-11-06

**Authors:** Daniel G. E. Thiem, Daniel Schneider, Michael Hammel, Bassam Saka, Bernhard Frerich, Bilal Al-Nawas, Peer W. Kämmerer

**Affiliations:** 1 Department of Oral and Maxillofacial Surgery, Facial Plastic Surgery, University Medical Centre Mainz, Augustusplatz 2, 55131 Mainz, Germany; 2 Department of Oral and Maxillofacial Surgery, Facial Plastic Surgery, University Medical Centre Rostock, Schillingallee 35, 18057 Rostock, Germany; 3 Oral, Maxillofacial and Plastic Surgery, Rostock, Germany; 4 Sanitz, Germany; 5 Department of Oral and Maxillofacial Surgery, School of Dentistry, Kyung Hee University, Seoul, Korea

**Keywords:** Patient information, Surgery, Side effect, Elective surgery, Complications, QoL, SF-36

## Abstract

**Objectives:**

The aim of this clinical analysis was to evaluate intraoperative and early postoperative complications as well as late findings and the overall patient satisfaction following orthognathic surgery.

**Materials and Methods:**

In a retrospective, cross-sectional study, 119 patients after orthognathic surgery were included. Surgical approaches were single bilateral sagittal split osteotomy (BSSO (*n* = 52)), single LeFort-I osteotomy (*n* = 5) and bimaxillary osteotomy (LeFort-I + BSSO (*n* = 62)). Intraoperative and early (0–4 weeks postoperative) complications were investigated retrospectively (*n* = 119), whereas late findings and quality of life were assessed via clinical follow-up and survey (mean: 59 months postoperative) on 48 patients.

**Results:**

Bad split (*n* = 4/114) was the most common intraoperative complication followed by one case of severe bleeding. Regarding early postoperative complications, temporary damage of the inferior alveolar nerve after BSSO was most common (*n* = 33/114), followed by facial nerve dysfunction (*n* = 3), failed osteosynthesis (*n* = 2) and one case of postoperative dyspnoea. Permanent hypaesthesia of the lower lip was the most prevalent (*n* = 28/45^(BSSO and LeFort-I + BSSO)^) late finding with varying extent, followed by temporomandibular dysfunction (TMD) (*n* = 25/48). Skeletal relapse mostly occurred after class II treatment, followed by class III, posterior crossbite and open bite. Overall, the surgery improved the patients’ self-perception (85.4%), with 60.4% of patients opting for surgery again.

**Conclusions:**

Long-term complications after orthognathic surgery occurred more frequently than commonly described in the literature, and analyses of the quality of life show the need for more comprehensive preoperative patient education.

**Clinical relevance:**

Hypaesthesia of the lower lip presented less as complication but rather as side effect following BSSO. As orthognathic surgery is mostly elective, preoperative patient education is of pivotal importance and should include proactive risk stratification.

## Introduction

Orthognathic surgery is a commonly used procedure to correct dentofacial deformities in patients who are too old for growth modification as well as for dentofacial conditions that are too severe for either surgical or orthodontic camouflage [1]. In this context, LeFort-I and bilateral sagittal split osteotomy (BSSO) are the most commonly used procedures [2, 3]. LeFort-I osteotomy was first described by Cheever in 1864 for the resection of a nasopharyngeal tumour but is named after the fracture pattern originally described by Rene LeFort in 1901 [4]. In 1957, Trauner and Obwegeser [5] described BSSO which was then modified by Dal Pont (1961), Hunsuck (1968) and Epker (1977) [6]. However, there is a wide variety of intraoperative (bleeding, inadequate osteotomy/bad split, nerve exposure and damage, dental injuries) and postoperative complications (persistent paresthesia due to nerve injuries, plate fractures, dyspnoea, skeletal relapse, temporomandibular joint dysfunction (TMD)) associated with orthognathic surgery [2, 7–9]. In particular, BSSO is related to inferior alveolar nerve (IAN) damages with subsequent numbness of the chin and the lower lip [10, 11]. In this context, one review summarised that nerve injuries occur most frequently (50%), followed by temporomandibular joint disorders (TMD) (14%), haemorrhage (9%) and relapse (4%) [1, 12]. TMD is a subgroup of craniomandibular dysfunction (CMD) but does not include occlusion as a criterion. As most patients undergo orthognathic surgery for aesthetic purposes, it is crucial that they receive detailed education on the general and special operative risks and the respective impact on their future health. Besides, patients who completed orthognathic surgery reported a large variety of psychological benefits, such as improved self-confidence and self-esteem [13]. However, dissatisfaction also arises as a result of unfulfilled patient expectations [14].

Therefore, the primary aim of the study was to evaluate intraoperative and early postoperative complications as well as late findings following orthognathic surgery. Secondary, this study investigated the overall long-term patient’s quality of life status after surgical treatment.

## Materials and methods

In a retrospective clinical cross-sectional study, 119 patients who underwent orthognathic surgery between January 2010 and June 2016 at a University Medical Department of Oral and Maxillofacial Surgery in Germany were included. The study was approved by the local ethics committee, Germany (registration number: A 2016-0171), and was conducted in accordance with the protocol and in compliance with the moral, ethical and scientific principles governing clinical research as set out in the Declaration of Helsinki of 1975 as revised in 1983.

### Surgical treatment

LeFort-I osteotomy and BSSO were both performed as described in detail for LeFort-I osteotomy by Buchanan and Hyman [4] and for BSSO by Monson [6]. The mandibular split osteotomy was performed according to Obwegesers’ surgical method modified by Dal Pont (Obwegeser/Dal Pont) [15]. In a subgroup analysis, surgeons were grouped according to their level of surgical experience (i.e. number of procedures) (beginner: < 10, intermediate: 10–40, experts: > 40) [16].

### Data collection on intraoperative and early complications

All analogue and digital data were obtained from the patients’ surgical and orthognathic records. Pre- and postoperative radiographs (orthopantomography (OPT and cone beam computed tomography (CBCT)) were analysed. The data extracted included patient specifics as date of birth, gender, diagnosis, medical history, in-patient time, comorbidities and diagnosis as well as the respective treatment. Information of interest regarding intraoperative and early postoperative complications was as follows:

- Intraoperative: bad split, haemorrhage, dental injuries
- Early (during 4 weeks) postoperative: delayed wound healing, failed osteosynthesis, nerval sensitivity disorders (e.g. hypaesthesia and anaesthesia), dyspnoea, pain/dysfunctions of temporomandibular joint (TMD).

The exclusion criteria of this study were incomplete medical records and missing data from the hospital’s database, patients with dentofacial trauma history, hemifacial microsomia, cleft lips, craniosynostosis, degenerative or inflammatory conditions and children younger than 16 years of age.

### Long-term follow-up—clinical analysis and late findings

**Fig. 1:**
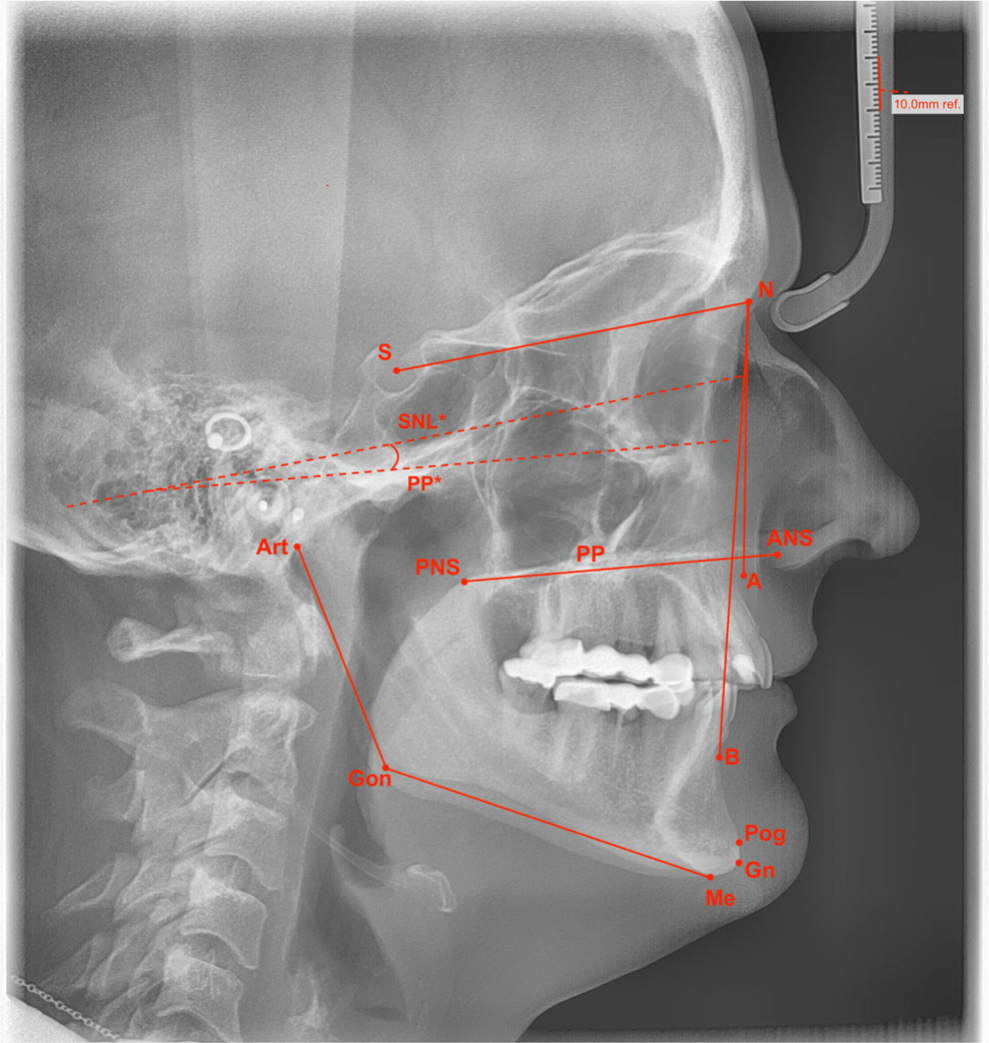
Lateral cephalometric radiograph with landmarks, lines and angles (red) serving as reference points. Landmarks are N, nasion; S, sella; A, A point (most concave point of anterior maxilla); B, B point (most concave point on mandibular symphysis); Pog, pogonion (most anterior point of mandibulae symphysis); Me, menton (lowest point on mandibular symphysis); Gn, gnathion (point of intersection between the line between pogonion and menton); Gon, gonion (point of intersection between ramus plane and mandibular plane); Art, articulare (intersection between the ascending ramus and the inferior surface of the cranial base); PNS, posterior nasal spine; ANS, anterior nasal spine, lines: SNL, sella-nasion line; PNS-ANS, maxillary length; Gn-Gon, mandibular plane; gonial angle, angle formed by a line connecting gnathion to gonion to articulare, and angles: SNA (°), SNA-angle (indicates whether or not the maxilla is normal, prognathic or retrognathic); SNB (°), SNB angle (indicates whether the mandible is normal, prognathic or retrognathic); ANB (°), ANB angle (indicates whether the relationship between maxilla and mandible is regular, a skeletal class II or class III. The patient has agreed in writing to the use of the X-ray image for publication.

In the second part of the study, patients were re-examined in a clinical follow-up in the average of 59 months (SD ± 19.7; min: 12; max: 84 months) after surgery. For this purpose, every included patient was contacted via letter or phone. In total, 48 patients (40.3%) did respond and were subsequently examined clinically. Late findings included persistent nerve damage (infraorbital (ION), lingual, inferior alveolar (IAN), buccal and facial nerve), dysfunction of the TMJ and masticatory musculature, i.e. arthropathy and myopathy on palpation (summarised as temporomandibular joint disorder (TMD)) and relapse. For nerve diagnostics, sensitivity was tested using cotton wool swabs followed by a pointed probe. Both were consecutively applied bilateral to the areas of interest, e.g. outer as well as the inner surface of the medial and lateral lower lip (branches of the mental nerve), cheek mucosa (buccal nerve), anterior and posterior gingiva of the upper (anterior, medial, posterior branches of the superior alveolar nerves) and lower jaw (sensory branches of the inferior dental plexus), upper lip and upper vestibular mucosa (branches of the infraorbital nerve) and tongue (left and right lingual nerve). No sensation was defined as anaesthesia whereas some superficial pain and touch sensation but reduced in comparison to the surrounding areas was stated hypaesthesia. The normal sensation was defined as a subjective normal test result. As the facial nerve supplies motor branches to the muscles of facial expression, functionality tests were conducted by asking the patient to crease up their forehead (temporal branches of the facial nerve), close their eyes (temporal and zygomatic branches of the facial nerve) and to puff out their cheeks and reveal their teeth (buccal and marginal mandibular branch of the facial nerve). Regarding TMD, the clinical examination included observation of the mouth opening pattern for deviation, direct bilateral preauricular palpation to assess condylar movements and pain sensitivity, provocation tests including joint traction in caudal and compression in dorso-cranial direction. Masticatory muscle disorders were examined for myalgia via direct palpation (temporal muscles, masseter muscles, medial and lateral pterygoid muscles, anterior and posterior belly of the digastric muscles). Here, myopathy was diagnosed if one muscle revealed pain. Relapse was measured based on lateral cephalometric radiographs (LCR) with a comparison between direct postoperative controls (t1) and LCR at follow-up (t2). Angle class II relapse (posterior relapse) was diagnosed if the SNB angle decreased more than 3° after BSSO and/or SNA-angle increased > 3° following LeFort-I + BSSO. Angle class III relapse (anterior relapse) was defined as an increase of SNB and/or an increase of SNA (> 3°). Relapse of a skeletal open bite was diagnosed if the SNL-PP (°)-angle between the sella-nasion line (SNL) and the palatal plane (PP) [anterior (ANS) to posterior (PNS) nasal spine)] was reduced > 3° from t1 to t2 (Fig. 1). Relapse of a posterior crossbite (inadequate transversal relationship of maxillary and mandibular teeth, i.e. the buccal cusps of the maxillary teeth, is in contact with the central fossae of the mandibular teeth) was diagnosed clinically by occlusion control. In addition, all patients received a questionnaire consisting of a modified form of the Medical Outcomes Study 36-Item Short-Form Health Survey (SF-36) [17, 18]. The study-specific questionnaire contained *n* = 18 questions (yes/no questions and numeric (1 = not at all, 6 = very much) response scale) addressing mental health, psychical and physical functioning, pain, functional limitations, satisfaction and general health.

### Statistics

The sample size (*n* = 119) is in accordance with the current literature [17, 19–21]. Raw data sets were saved in Excel® sheets (Microsoft Corporation, Redmond, USA) and subsequently transferred into SPSS Statistics® version 23.0.0.2 (MacOS X; SPSS Inc., IBM Corporation, Armonk, NY, USA). Data were expresses as median (MD), mean (m), standard deviation (SD ±), minimum (min) and maximum (max). Normal distribution was checked using the non-parametric Kolmogorov-Smirnov test (KS test) and results were analysed for statistical significance by the use of analysis of variance (ANOVA), unpaired non-parametric Mann-Whitney *U* tests, Wilcoxon Whitney tests and Students’ *t* test. To compare binary data of two or more unpaired samples, chi-square test^(§)^ was used. Pearson correlation^(##)^ was used to measure the strength and direction of linear relationships between pairs of continuous variables, whereby categorical variables were recoded as 0 and 1. A *p* value < 0.05 was considered significant.

## Results

### Age and gender of patients and experience of the surgeon

In this study, 55.5% (*n* = 66) of patients were women and 44.5% (*n* = 53) were men, with a total mean age of 31.3 years (SD: ± 11.1; MD: 28.0; min: 17.0; max: 62.0). Seventy-three out of one hundred nineteen patients were treated by an experienced surgeon (> 40 orthognathic surgeries), 31 by an intermediate-skilled surgeon (10–40 orthognathic surgeries) and 15 patients by a beginner (< 10 orthognathic surgeries).

### Diagnosis, treatment and in-patient time

With *n* = 75, angle class II malocclusion was the most frequent leading diagnosis, followed by angle class III (*n* = 39), posterior crossbite (*n* = 3) and frontal open bite (*n* = 2). Combined diagnoses were angle class II^(leading)^ + posterior crossbite, angle class II^(leading)^ + frontal open bite, angle class III^(leading)^ + frontal open bite, angle class III^(leading)^ + crossbite, posterior crossbite^(leading)^ + angle class III and open bite + angle class II (Table 1). The most frequently performed treatment was a LeFort-I osteotomy + BSSO (*n* = 62), followed by single BSSO (*n* = 52) and single LeFort-I osteotomy (*n* = 5) (Table 1). The average in-patient time was 7.9 days (SD ± 2.1, MD: 8.0, min: 4, max: 19 days).

**Table 1:** Distribution of patients for diagnosis and operation techniques

	Percentage (%)	Total (*n*)
Leading diagnosis		
Angle class II	63.0	75
Angle class III	32.8	39
Posterior crossbite	2.5	3
Frontal open bite	1.7	2
Total	100.0	119
Combined diagnosis		
Angle class II		
+ Posterior crossbite	3.4	4
+ Frontal open bite	2.5	3
Angle class III		
+ Posterior crossbite	3.4	4
+ Frontal open bite	1.7	2
Posterior crossbite		
+ Angle class III	0.8	1
Total	11.8	14/119
Treatment		
Single LeFort-I	4.2	5
Single BSSO	43.7	52
LeFort-I + BSSO	52.1	62
Total	100.0	119

### Intraoperative complications

Out of the overall five intraoperative complications, bad splits were most frequented (4/114), showing an incidence of 3.5% per BSSO. Related to 228 split osteotomies (SSO = BSSOx2), the incidence of the bad split was 1.7% per SSO. Furthermore, one case of intraoperative haemorrhage occurred during a single LeFort-I osteotomy (Table 2).

**Table 2:** Overview of intraoperative and early complications as well as late findings

	Percentage (%)	Total (*n*)
Intraoperative complications		
Bad split (SSO)	3.5 (1.7)	4/114 (4/228 SSO)
Bleeding (with intervention)	0.8	1/119
Total		5/119
Early complications and adverse events (a.e.*)		
Sensitivity disorder	31.6	36/114^(BSSO + LeFort-I + BSSO)^
Hypaesthesia		
• Lower lip (inferior alveolar nerve)	28.9	33
Anaesthesia		
• Medial lower lip left (mental nerve)	2.6	3
Delayed wound healing	/	/
TMJ sensation	/	/
Failed osteosynthesis (*)	1.7	2/119
Dyspnoea	0.8	1/119
Late findings		
Relapse after surgical treatment of:^#^	25.0	12/48 (cases)
• Angle class II (posterior relapse; (*n* = 31)*)	66.6	8/12
• Single LeFort-I	/	/
• Single BSSO (*n* = 21)*	75	6/8
• LeFort-I + BSSO (*n* = 10)*	25	2/8
• Angel class III (anterior relapse; (*n* = 15)*)	16.6	2/12
• Single LeFort-I (*n* = 3)*	100	1/1
• Single BSSO (*n* = 2)*	/	/
• LeFort-I + BSSO (*n* = 10)*	/	/
• Posterior crossbite + angle class III (*n* = 1)*	8.3	1/12
• Single LeFort-I	/	/
• Single BSSO	/	/
• LeFort-I + BSSO	100	1/1
• Frontal open bite + angle class II (*n* = 1)*	8.3	1/12
• Single LeFort-I	/	/
• Single BSSO	/	/
• LeFort-I + BSSO	100	1/1
TMD arthropathy^#^	29.2	14/48 cases
• Single BSSO	26.1	6/23
• LeFort-I + BSSO	36.4	8/22
TMD myopathy^#^	31.2	15/48 cases
• Single BSSO	39.1	9/23
• LeFort-I + BSSO	27.3	6/22
TMD total^#^	52.1	25/48
Permanent anaesthesia—area of the lower lip (medial left)	2.2 (1.1)	1/45 (1/90 SSO)
Permanent hypaesthesia—areas on the lower lip (90 SSO)	62.2 (31.1)	28/45 (28/90 SSO)
• Lateral lower lip left	11.1	5/45
• Lateral lower lip right	11.1	5/45
• Medial lower lip left	42.2	19/45
• Medial lower lip right	40.0	18/45
• Medial + lateral aspect of left lower lip	8.8	4/45
• Medial + lateral aspect of right lower lip	6.6	3/45
• Complete lower lip (left and right side)	2.2	1/45
Permanent hypaesthesia—areas on the upper lip (infraorbital nerve)	8	2/25 cases
• Single LeFort-I	33.3	1/3
• LeFort-I + BSSO	4.5	1/22
Permanent hypaesthesia tongue (lingual nerve right and left)	/	/
Permanent hypaesthesia cheek mucosa (left buccal nerve after BSSO)	2.1	1/48 cases

Events that are considered undesirable rather than complications were marked with (^#^). SSO stands for sagittal split osteotomy and is performed twice (right and left) in one BSSO. The total number of diagnosis and surgical procedures at long-term follow-up is indicated by (*)

### Early complications

A deficiency of the mandibular nerve sensitivity was the most common early postoperative complication. Overall 33 patients (27.7%) presented with hypaesthesia and three patients (2.5%) with anaesthesia. Information on site distribution could not be obtained from the clinical documentation. Further early complications were two cases of failed osteosynthesis (1.7%) and one case of postoperative dyspnoea (0.8%). There was no case with impaired postoperative wound healing, secondary bleeding or TMJ complaints.

### Late findings and clinical follow-up

Late findings were present in overall 46 of 48 follow-up cases and were subgrouped into myopathy (*n* = 15/48), arthropathy (*n* = 14/48), relapse (*n* = 12/48) and permanent sensitivity disorders of the lower lips (*n* = 28/45^(BSSO and LeFort-I + BSSO)^) (Table 2). Myopathy was present in 9/23 cases of single BSSO and overall 6/22 cases of LeFort-I + BSSO. Arthropathy was revealed in 6/23 single BSSO cases and in 8/22 cases following LeFort-I + BSSO. Skeletal relapse occurred in eight cases after the treatment of angle class II, two patients with angle class III, one with posterior crossbite and one patients with a frontal open bite (Table 3). Long-term examination of the perioral sensitivity revealed 28/45^(BSSO and LeFort-I + BSSO)^ patients with sensitivity disorders of the lower lip (one single area measured with anaesthesia and 46 areas with hypaesthesia) (Table 2). In detail, there were five patients showing isolated hypaesthesia of the lateral supply area of the mental nerve/IAN (lateral aspect of the lower lip), whereas isolated hypaesthesia of the lower lips’ medial aspect was present in 27 patients. Hypaesthesia on both, the medial and lateral aspect, occurred in overall six patients (left = 4; right = 3) with one case affecting the entire lower lip. Hypaesthesia of the upper lip (supply area of the ION) was present in one patient on the right side following LeFort-I + BSSO as well as in one case following single LeFort-I osteotomy (left side). There was no neurosensory deficit of the lingual nerve. For the buccal nerve, one case presented with hypaesthesia of the left cheek mucosa after a single BSSO (Table 2). For myopathy, arthropathy and permanent nerve damages, there was no significant correlation to the surgical approach (myopathy: *p* = 0.33^(§)^, arthropathy: *p* = 0.39^(§)^, permanent nerve damages (IAN: *p* = 0.58^(§)^). In contrast, the number of skeletal relapses was significantly higher for BSSO and LeFort-I + BSSO in comparison to single LeFort-I osteotomy (*p* = 0.007^(§)^).

**Table 3:** Complications according to surgeons’ experience

	Surgeons’ experience			
	Beginner	Intermediate	Expert	*p* value
Intraoperative complications				
Bad split	1/15	3/30	0/69	0.035
Dental injuries	-	-	-	-
Bleeding/transfusion	0/15	1/31	0/73	0.24
Early complications				
Nerve dysfunction	7/15	9/31	20/73	0.55
TMJ pain	0/15	0/31	0/73	-
Failed osteosynthesis (^#^)	0/15	0/31	2/73	-
Delayed wound healing	0/15	0/31	0/73	-
Respiratory dysfunction	0/15	0/31	1/73	0.73
Late findings (*n* = 48 follow-ups)				
Relapse				
• Angle class II	3	2	8	0.59
• Angle class III	3	1	4	0.21
• Posterior	0	1	1	0.79
• Crossbite	0	0	1	0.67
• Frontal open bite	0	0	2	0.46
Arthropathy	2/8	4/13	8/27	0.96
Myopathy	2/8	3/13	10/27	0.72
Persistent nerve damage lower lip	6/8	8/13	14/24	0.51

Surgeons’ experience: beginner (< 10 orthognathic operations), intermediate (10–40 orthognathic operations), experts (> 40 orthognathic operations)

Events that are considered undesirable rather than complications were marked with (^#^)

### Associations between clinical findings and the surgeons’ level of experience

Regarding bad splits as an intraoperative complication, there was a significant correlation (*p* = 0.035^(§)^) between the surgeons’ experience and the frequency of events [beginner: 1/15, intermediate: 3/31 and experienced: 0/73]. In this study, overall 36 cases of early nerve dysfunction (no site differentiation) have been observed. For beginners, *n* = 7/15 cases presented with nerval disorders, whereas intermediate surgeons accounted for *n* = 9/31 and experienced surgeons for *n* = 20/73 of cases. There was no significant correlation between the surgeons’ experience and the rate of sensitivity disorders in the early postoperative phase (*p* = 0.55^(§)^). Failed osteosynthesis by means of plate loosening was present in two cases, whereby both were operated by an expert (*p* = 0.47). No significant correlation was found between the surgeons’ expertise and the frequency of late postoperative findings [myopathy (*p* = 0.72), arthropathy (*p* = 0.96), relapse (*p* = 0.59), dysaesthesia of the lower lip (*p* = 0.51)] (Table 3).

### Quality of life (SF-36)

Frequency and distributions of numerical variables are shown in (Appendix Table 4). At the time of questioning, most patients (39.6%) rated their general health status as very good. Compared to the preoperative assessment, most (39.6%) patients remembered no difference. In the first four postoperative weeks, patients felt physical restrictions (60.4%) more causative for a limited performance than mental issues (14.6%). Most patients decided for surgery due to aesthetic reasons (60.4%), followed by limited chewing function (14.6%), the influence of the partner (4.2%), the parents (4.2%), friends (2.1%) and the dentist (14.6%). Postoperatively, patients (33.3%) and their relatives (37.5%) were mostly satisfied with the overall surgical outcome. In addition, 13 patients (27.1%) remembered the pain in the temporomandibular joint preoperatively, whereas ten patients (20.8%) described postoperative TMJ pain. With regard to snoring, patients did not observe any changes postoperatively (54.2%_preoperative_ and 52.1%_postoperative_). In total, *n* = 29/48 (60.4%) of patients did not regret their decision upon surgery and would opt for surgery again. In contrast, 25% of patients were not sure and only 14.6% would not repeat the procedure.

## Discussion

Considering the selectivity of orthognathic surgery [4, 6], knowledge about the common complications and the overall patient outcome is of paramount importance for sufficient preoperative patient information. The aim of this study is to evaluate the rate of perioperative complications following orthognathic surgery as well as to assess the patient’s quality of life in a long-term follow-up. The special feature of the present study is the long follow-up period of 59 months on average in connection with the assessment of the patients’ quality of life and the detailed clinical examination with a focus on the perioral sensory system.

An irregular fracture of the mandible during the course of BSSO is termed a bad split, showing an incidence of 1 to 23% [22]. This retrospective analysis revealed overall four bad splits in 114 patients, presenting an incidence of the bad split of 1.7% per SSO. This is in accordance with a literature review and meta-analysis by Verweij et al. who reported a pooled incidence of the bad split of 2.3%, whereas the included studies’ incidence varied between 0.5 and 14% per SSO [23]. In addition, this study revealed a significant (*p* = 0.035^(§)^) relationship between the surgeons’ expertise and the occurrence of bad splits (beginner and intermediate: 3 versus experts: 0) which is in line with other studies [24, 25]. Al-Nawas et al. reported more bad splits when BSSO was performed by inexperienced surgeons, although they did not show statistically significant differences [16]. Other reported risk factors for bad splits are a higher age, presence, position and root morphology of mandibular third molars, mandibular morphology and osteotomy design [23]. However, most studies, including this, are sharing the drawback of no standardisation with respect to the surgeon. Apart from bad splits, haemorrhage with subsequent blood transfusion was the second type of intraoperative complication in this study and was present in one patient following a single LeFort-I osteotomy. This is in contrast to the literature, reporting 9% of haemorrhage following LeFort-I osteotomy as a consequence of pterygomaxillary separation [26, 27]. However, a systematic review by Pineiro-Aguilar et al. has shown that the intraoperative blood loss in patients during orthognathic surgery (single LeFort-I or BSSO and LeFort-I + BSSO) is less than the limits set for blood transfusion [28]. Hence, with one case only, the present result should be rated as an exception.

With regard to early postoperative findings, hyp- (*n* = 33) and anaesthesia (*n* = 3) of the lower lip, indicating damage of the inferior alveolar nerve, was most common in this study (*n* = 36/114^(BSSO and LeFort-I + BSSO)^, 31.6%). In literature, the frequency of neurosensory deficits ranges from 8 to 32% [2], which is in line with this study’s early postoperative results. During the early postoperative phase, patients often observed sensitivity disorders or at least any kind of numbness within the innervation area of the inferior alveolar nerve, whereas 6 months to 1 year are considered regular for complete recovery [2]. This contrasts with the present results, in which a total of 28/45 (62.2%) patients with at least permanent partial hypaesthesia of the lower lip (IAN damage) after mandibular osteotomy and 2/25 (8%) patients with partial but permanent hypaesthesia of the upper lip after LeFort-I osteotomy (including individual LeFort-I and LeFort-I + BSSO) were found in long-term follow-up (> 12 months after surgery). In this context, however, it is important to emphasise the fact that these are examinations during the immediate postoperative period and the first four postoperative weeks, which involves a change of examiners and documentation of varying detail. A probable cause for the percentage increase in sensory deficits from early postoperative to long-term follow-up can therefore be assumed to be the inhomogeneous, false-negative examination documentation, which is not least a general disadvantage of retrospective data evaluation in general.

Regarding any affection of the temporomandibular joint and its adjacent musculature (TMD) as late findings, this study revealed several restrictions (*n*_arthropathy_ = 14; *n*_myopathy_ = 15) following orthognathic surgery. In general, this is supported by literature, reporting pain on palpation upon TMJ and the masticatory musculature as a common diagnosis [29–32]. However, due to different surgical methods and follow-up periods, results are not directly comparable. Some patients’ postoperatively suffered from TMJ dysfunction by deformation of the condylar surface, condylar resorption and malocclusion as a result of condylar sag, whereas others reported favourable effects on the TMJ function following surgery [1]. As joint degeneration develops over a long period, any influence of orthognathic surgery on the TMJ cannot be clarified yet. Hence, more in-depth studies with MRI-based follow-ups are needed to determine the full effect. Skeletal stability after orthognathic surgery is controversially discussed in the literature and it was shown that postsurgical relapse mainly develops within the first 6 months after surgery [33]. Late relapse on the other hand tends to develop from 6 to 12 months after surgery and is believed to occur due to individual patient characteristics, such as condylar resorption as well as type, direction and magnitude of surgical displacements [34, 35]. However, little is known about long-term stability after orthognathic surgery. In the long term, this study showed overall 25.0% (*n* = 12/48) of skeletal relapse after 59 months on average, with most cases occurred after surgical treatment of class II malocclusion (*n* = 8/12) using single BSSO (*n* = 6/8) and more rarely, LeFort-I + BSSO (*n* = 2/8). This is supported by other studies, showing that large surgical advancements of the mandible (> 6 to 7 mm) are more prone to relapse and is explained by an increased tension of soft tissue and musculature [36, 37]. On the other hand, other authors previously described bimaxillary surgery as less stable in patients with a class II malocclusion than single BSSO [38, 39]. For class III treatment, De Haan et al. found both bimaxillary and single BSSO as equivalent regarding skeletal stability [40]. In this study, only two cases of a class III^(leading)^ relapse was observed; thus, valid statements are not possible. The same applies to recurrences of a frontal open bite^(leading)^ (*n* = 1) and posterior crossbite^(leading)^ (*n* = 1) as well. However, the literature references on this subject are inhomogeneous regarding the extent and method of measurement. While in one study, the change of B and Pog are measured in millimetre; others refer to changes of cephalometric angles (e.g. ANB) [41]. Furthermore, there are differences in the same measurement, depending on the imaging modality used (2D or 3D), with differences averaging > 1 mm [42]. Another influencing factor is the timepoint of the postoperative control (T2/baseline). In this study, T2 was performed directly after surgery; other authors refer to a postoperative control x-ray 8 weeks after surgery. At this point, in most cases, the final splint is removed and the mostly hypertonic muscles are already actively exerting force [41]. In summary, the radiograph at T2 already represents a status deviating from the originally planned target position, so that a statement regarding the deviation from T3 is only possible to a limited extent. Ultimately, however, there is no consistent classification for skeletal relapse following orthognathic surgery and therefore no existing thresholds. Regarding IAN damages, our results are in line with a systematic review by Phillips and Essick, showing that 60% of patients had hypaesthesia in the supply area of IAN after 2 years [43]. Mandibular osteotomies (BSSO) are performed in close proximity to the neurovascular bundle, and the placement of semi-rigid fixation plates and screws may additionally cause nerve damage either directly or indirectly via nerve compression between the bony segments after screw fixation. Hu et al. observed no difference in the number of nerve damages when using either bicortical or monocortical osteosynthesis [44]. For ION, Thygesen et al. found subjective changes in somatosensory function after LeFort-I osteotomy in 7 to 60% of patients after a 1-year follow-up [43, 45].

Generally, the surgeons’ experience seems to influence the patient’s outcome in various treatments of maxillofacial surgery [46]. In this context, Al-Nawas et al. [16] showed marginal but not significant associations between less experienced surgeons and the frequency of bad splits, intraoperative bleeding and cases of delayed wound healing, whereas plate loosening and fractures and nerve injuries were more common in the expert group. This is consistent with the available results, with the total number of intraoperative and early complications and late findings for each group of surgeons in this study being *n* = 15 for beginners, *n* = 23 for advanced and *n* = 49 for experts. However, surgeons with intermediate training and experts performed more operations, so that the proportion of complications decreased with increasing experience (beginners): 15/15 (100%), advanced: 23/31 (74.2%), experts: 49/73 (67.1%)), with no significant correlation between the complication rate and the surgical competence level. However, intermediate trained surgeons and experts performed more operations; thus, the proportion of complications decreased with the level of experience (beginner: 15/15 (100%), intermediate: 23/31 (74.2%), experts: 49/73 (67.1%)), with no significant correlation between the complication rate and the surgical skill level.

The most important reasons why patients decided upon orthognathic surgery were an improvement in aesthetics as well as the correction of functional impairments [47]. However, quality of life, especially psychological health, should be taken into consideration as well [48, 49]. An accepted tool to measure the general quality of life is the Medical Outcomes Study 36-Item Short-Form Health Surveys (SF-36) [17, 18]. In this study, a modified form of the SF-36 was used [50], showing the quality of life enhancement following orthognathic surgery. This is in accordance with the current literature, showing the general improved quality of life in patients who underwent orthognathic surgery [51–56]. In particular, the present study revealed that patients stated to have improved health conditions following surgery in the long term (45.9%) (Appendix Table 4). Postoperative pain seemed no major complaint in orthognathic surgery, while only seven patients rated their level of pain as severe or very severe. In literature, the psychological burden of patients with angle class II and III revealed similar without significant differences [57, 58].

One limitation of this study is the small number of long-term follow-up patients (*n* = 48), with an immanently limited significance of the results. Furthermore, the questionnaires were answered anonymously by the patients, which means that no correlation could be made between the results of the survey and the clinical findings. Due to different investigation time points, no temporal connection between the patients’ health status (physically or mentally) and the time after surgery could be found. As another drawback of this study, it has to be noted that the sensory innervation of the mental nerve may have some small overlap with the contralateral mental nerve, which makes full discrimination impossible [59]. TMJ and musculature palpation (pressure force) depended on the examiner and was therefore not standardised. At last, there was no control group of healthy patients.

## Conclusion

The number of severe complications in orthognathic surgery is low. However, (partial)-hypaesthesia of the lower lip occurred often in this study but is commonly underrepresented in the patients’ information before mandibular osteotomy. Hence, hypaesthesia of the lower lip seems to be less a complication but rather a regular consequence following BSSO. Despite a not negligible percentage of satisfied patients, orthognathic surgery improved the quality of life of the group of patients studied. In this context, comprehensive preoperative patient education with mention of the regularly occurring accompanying symptoms and recurrence rates is indispensable. To enhance study comparability, large controlled prospective studies are necessary.

## Appendix

**Table 4:** Evaluation of the SF-36 health survey questionnaire

Question Nr.	Question	Answer	n/48	%
1	General health: How would you rate your health in general?	Excellent	12	25.0
		Very good	19	39.6
		Good	13	27.1
		Fair	4	8.3
		Poor	0	0
2	General health: Compared to prior surgery, how would you rate your health in general now?	Much better now than before the surgery	9	18.8
		Somewhat better now than before the surgery	13	27.1
		About the same	19	39.6
		Somewhat worse now than before the surgery	6	12.5
		Much worse than before the surgery	1	2.1
3	Physical Health Problems Have you had any of the following problems regarding your work or other regular daily activities as a result of your physical health during the four weeks after surgery?			
	I became tired earlier than usual.	Yes	26	54.2
		No	22	45.8
	I accomplished less than I normally would.	Yes	29	60.4
		No	19	39.6
	I was limited to certain work or activities.	Yes	35	72.9
		No	13	27.1
	I had difficulties with the execution at work or other activities (e.g. it required a special effort)	Yes	29	60.4
		No	19	39.6
4	Mental Health Problems Have you had any of the following problems regarding your work or other regular daily activities as a result of your mental health during the four weeks after surgery?			
	I became tired earlier than usual.	Yes	8	16.7
		No	40	83.3
	I accomplished less than I normally would.	Yes	7	14.6
		No	41	85.4
	I was not able to work as careful as I normally do.	Yes	10	20.8
		No	38	79.2
5	Impact on the social environment After four weeks following the surgery, how much did your physical and/or mental health situation influenced the relationship between you and your family, friends and neighbors?	Not at all	10	20.8
		Very slightly	7	14.6
		Slightly	14	29.2
		Moderately	11	22.9
		Severe	5	10.4
		Very Severe	1	2.1
6	Pain During the past 4 weeks, how much did pain interfere with your normal work (including both work outside the home and housework)?	Not at all	11	22.9
		Slightly	17	35.4
		Moderately	13	27.1
		Severe	6	12.5
		Very Severe	1	2.1
7	Motivation What was your incentive reason to undergo surgery?	Aesthetics	29	60.4
		Chewing function	7	14.6
		Speech	0	0.0
		Snoring	0	0.0
		Influence of the partner	2	4.2
		Influence of the parents	2	4.2
		Influence of the friends	1	2.1
		Influence of the dentist/medical doctor	7	14.6
8	Satisfaction 1 From an aesthetical point of view, how satisfied are you with the surgical result?	Excellent	12	25.0
		Very good	13	27.1
		Good	16	33.3
		Fair	7	14.6
		Poor	0	0.0
9	Satisfaction 2 From an aesthetical point of view, how satisfied is your next environment (partner/parents) with the surgical result?	Excellent	16	33.3
		Very good	9	18.8
		Good	18	37.5
		Fair	5	10.4
		Poor	0	0.0
10	Satisfaction Do you find your look more favorable now than before the surgery?	More favorable	20	41.7
		Some favorable	25	52.1
		Some unfavorable	2	4.2
		More unfavorable	1	2.1
11	Functional 1 Did you have pain in the temporomandibular joint before the surgery?	Spontaneous	4	8.3
		In case of physical stress (chew)	4	8.3
		In case of mental stress	2	4.2
		Weather changes	3	6.3
		No	35	72.9
12	Functional 2 Did you develop pain in the temporomandibular joint after the surgery?	No	37	77.1
		Yes, the same as before	6	12.5
		Yes, but easier	4	8.3
		Yes, but intense	1	2.1
13	Functional 3 Did you observed a change in the mobility of the mandible since the surgery?	No	37	77.1
		Yes, significantly more restricted	0	0.0
		Yes, more restricted	3	6.3
		Yes, significantly more mobile	1	2.1
		Yes, more mobile	7	14.6
14	Physical Health Problems Have you had daytime sleepiness before the surgery?	No	0	0.0
		Yes, seldom	4	8.3
		Yes, sometimes	10	20.8
		Yes, regularly	34	70.8
15	Physical Health Problems Do you have daytime sleepiness since the surgery?	No	1	2.1
		Yes, seldom	3	6.3
		Yes, sometimes	11	22.9
		Yes, regularly	33	68.8
16	Physical Health Problems Have you been snoring before the surgery?	No	4	8.3
		Yes, seldom	9	18.8
		Yes, sometimes	13	27.1
		Yes, regularly	22	45.8
17	Physical Health Problems Do you snore since the surgery?	No	2	4.2
		Yes, seldom	8	16.7
		Yes, sometimes	15	31.3
		Yes, regularly	23	47.9
18	From today’s perspective, would you decide to undergo the surgery again?	Yes	29	60.4
		No	7	14.6
		I’m not sure	12	25.0

## References

[1] Jedrzejewski M, Smektala T, Sporniak-Tutak K, Olszewski R (2015). Preoperative, intraoperative, and postoperative complications in orthognathic surgery: a systematic review. Clin Oral Investig.

[2] Iannetti G, Fadda TM, Riccardi E, Mitro V, Filiaci F (2013). Our experience in complications of orthognathic surgery: a retrospective study on 3236 patients. Eur Rev Med Pharmacol Sci.

[3] Thiele OC, Kreppel M, Bittermann G, Bonitz L, Desmedt M, Dittes C, Dorre A, Dunsche A, Eckert AW, Ehrenfeld M, Fleiner B, Frerich B, Gaggl A, Gerressen M, Gmelin L, Hammacher A, Hassfeld S, Heiland M, Hemprich A, Hidding J, Holzle F, Howaldt HP, Iizuka T, Kater W, Klein C, Klein M, Kohnke RH, Kolk A, Kubler AC, Kubler NR, Kunkel M, Kuttenberger JJ, Kreusch T, Landes C, Lehner B, Mischkowski RA, Mokros S, Neff A, Nkenke E, Palm F, Paulus GW, Piesold JU, Rasse M, Rodemer H, Rothamel D, Rustemeyer J, Sader R, Scheer M, Scheffler B, Schippers C, Schliephake H, Schmelzeisen R, Schramm A, Spitzer WJ, Stoll C, Terheyden H, Weingart D, Wiltfang J, Wolff KD, Ziegler CM, Zoller JE (2016). Moving the mandible in orthognathic surgery - A multicenter analysis. J Craniomaxillofac Surg.

[4] Buchanan EP, Hyman CH (2013). LeFort I Osteotomy. Semin Plast Surg.

[5] Trauner R, Obwegeser H (1957). The surgical correction of mandibular prognathism and retrognathia with consideration of genioplasty. I. Surgical procedures to correct mandibular prognathism and reshaping of the chin. Oral Surg Oral Med Oral Pathol.

[6] Monson LA (2013). Bilateral sagittal split osteotomy. Semin Plast Surg.

[7] Klemm E, Stosslein F, Murbe B (2001). Arteriovenous fistula of the maxillary artery, eustachian tube dysfunction and tinnitus after Le Fort I osteotomy. HNO.

[8] Kramer FJ, Baethge C, Swennen G, Teltzrow T, Schulze A, Berten J, Brachvogel P (2004). Intra- and perioperative complications of the LeFort I osteotomy: a prospective evaluation of 1000 patients. J Craniofac Surg.

[9] Jang SY, Kim MK, Choi SM, Jang JW (2013). Nasolacrimal duct obstruction after maxillary orthognathic surgery. J Oral Maxillofac Surg.

[10] Dodson TB (2011). Blood loss following orthognathic surgery varies widely and sometimes transfusions are needed. J Evid Based Dent Pract.

[11] Baas EM, Bierenbroodspot F, de Lange J (2015). Skeletal stability after bilateral sagittal split osteotomy or distraction osteogenesis of the mandible: a randomized clinical trial. Int J Oral Maxillofac Surg.

[12] Kim YK (2017). Complications associated with orthognathic surgery. J Korean Assoc Oral Maxillofac Surg.

[13] Baherimoghaddam T, Oshagh M, Naseri N, Nasrbadi NI, Torkan S (2014). Changes in cephalometric variables after orthognathic surgery and their relationship to patients’ quality of life and satisfaction. J Oral Maxillofac Res.

[14] Cheng LH, Roles D, Telfer MR (1998). Orthognathic surgery: the patients’ perspective. Br J Oral Maxillofac Surg.

[15] Dal Pont G (1961). Retromolar osteotomy for the correction of prognathism. J Oral Surg Anesth Hosp Dent Serv.

[16] Al-Nawas B, Kammerer PW, Hoffmann C, Moergel M, Koch FP, Wriedt S, Walter C (2014). Influence of osteotomy procedure and surgical experience on early complications after orthognathic surgery in the mandible. J Craniomaxillofac Surg.

[17] Nicodemo D, Pereira MD, Ferreira LM (2008). Effect of orthognathic surgery for class III correction on quality of life as measured by SF-36. Int J Oral Maxillofac Surg.

[18] Lee S, McGrath C, Samman N (2008). Impact of orthognathic surgery on quality of life. J Oral Maxillofac Surg.

[19] Cunningham SJ, Hunt NP, Feinmann C (1996). Perceptions of outcome following orthognathic surgery. Br J Oral Maxillofac Surg.

[20] Forssell H, Finne K, Forssell K, Panula K, Blinnikka LM (1998). Expectations and perceptions regarding treatment: a prospective study of patients undergoing orthognathic surgery. Int J Adult Orthodon Orthognath Surg.

[21] Rustemeyer J, Martin A, Gregersen J (2012). Changes in quality of life and their relation to cephalometric changes in orthognathic surgery patients. Angle Orthod.

[22] Veras RB, Kriwalsky MS, Hoffmann S, Maurer P, Schubert J (2008). Functional and radiographic long-term results after bad split in orthognathic surgery. Int J Oral Maxillofac Surg.

[23] Verweij JP, Houppermans PN, Gooris P, Mensink G, van Merkesteyn JP (2016). Risk factors for common complications associated with bilateral sagittal split osteotomy: a literature review and meta-analysis. J Craniomaxillofac Surg.

[24] Reyneke JP, Tsakiris P, Becker P (2002). Age as a factor in the complication rate after removal of unerupted/impacted third molars at the time of mandibular sagittal split osteotomy. J Oral Maxillofac Surg.

[25] Doucet JC, Morrison AD, Davis BR, Gregoire CE, Goodday R, Precious DS (2012). The presence of mandibular third molars during sagittal split osteotomies does not increase the risk of complications. J Oral Maxillofac Surg.

[26] O’Regan B, Bharadwaj G (2007). Prospective study of the incidence of serious posterior maxillary haemorrhage during a tuberosity osteotomy in low level Le Fort I operations. Br J Oral Maxillofac Surg.

[27] Politis C (2012). Life-threatening haemorrhage after 750 Le Fort I osteotomies and 376 SARPE procedures. Int J Oral Maxillofac Surg.

[28] Pineiro-Aguilar A, Somoza-Martin M, Gandara-Rey JM, Garcia-Garcia A (2011). Blood loss in orthognathic surgery: a systematic review. J Oral Maxillofac Surg.

[29] Panula K, Somppi M, Finne K, Oikarinen K (2000). Effects of orthognathic surgery on temporomandibular joint dysfunction. A controlled prospective 4-year follow-up study. Int J Oral Maxillofac Surg.

[30] Dervis E, Tuncer E (2002). Long-term evaluations of temporomandibular disorders in patients undergoing orthognathic surgery compared with a control group. Oral Surg Oral Med Oral Pathol Oral Radiol Endod.

[31] Abrahamsson C, Ekberg E, Henrikson T, Bondemark L (2007). Alterations of temporomandibular disorders before and after orthognathic surgery: a systematic review. Angle Orthod.

[32] Te Veldhuis EC, Te Veldhuis AH, Bramer WM, Wolvius EB, Koudstaal MJ (2017). The effect of orthognathic surgery on the temporomandibular joint and oral function: a systematic review. Int J Oral Maxillofac Surg.

[33] Yang HJ, Hwang SJ (2012). Postoperative stability following maxillary downward movement with Le Fort I inclined osteotomy at the lateral nasal cavity wall. J Craniomaxillofac Surg.

[34] Xi T, de Koning M, Berge S, Hoppenreijs T, Maal T (2015). The role of mandibular proximal segment rotations on skeletal relapse and condylar remodelling following bilateral sagittal split advancement osteotomies. J Craniomaxillofac Surg.

[35] Liebregts J, Baan F, van Lierop P, de Koning M, Berge S, Maal T, Xi T (2019). One-year postoperative skeletal stability of 3D planned bimaxillary osteotomies: maxilla-first versus mandible-first surgery. Sci Rep.

[36] Epker BN, Wessberg GA (1982). Mechanisms of early skeletal release following surgical advancement of the mandible. Br J Oral Surg.

[37] Van Sickels JE, Dolce C, Keeling S, Tiner BD, Clark GM, Rugh JD (2000). Technical factors accounting for stability of a bilateral sagittal split osteotomy advancement: wire osteosynthesis versus rigid fixation. Oral Surg Oral Med Oral Pathol Oral Radiol Endod.

[38] Proffit WR, Turvey TA, Phillips C (2007). The hierarchy of stability and predictability in orthognathic surgery with rigid fixation: an update and extension. Head Face Med.

[39] Brandtner C, Hachleitner J, Rippel C, Krenkel C, Gaggl A (2015). Long-term skeletal and dental stability after orthognathic surgery of the maxillo-mandibular complex in Class II patients with transverse discrepancies. J Craniomaxillofac Surg.

[40] de Haan IF, Ciesielski R, Nitsche T, Koos B (2013). Evaluation of relapse after orthodontic therapy combined with orthognathic surgery in the treatment of skeletal class III. J Orofac Orthop.

[41] Schwartz K, Rodrigo-Domingo M, Jensen T (2016). Skeletal stability after large mandibular advancement (> 10 mm) with bilateral sagittal split osteotomy and skeletal elastic intermaxillary fixation. J Oral Maxillofac Res.

[42] Sun Y, Tian L, Luebbers HT, Politis C (2018). Relapse tendency after BSSO surgery differs between 2D and 3D measurements: a validation study. J Craniomaxillofac Surg.

[43] Phillips C, Essick G (2011). Inferior alveolar nerve injury following orthognathic surgery: a review of assessment issues. J Oral Rehabil.

[44] Hu J, Zhao Q, Tang J, Zheng Z, Qi MC (2007). Changes in the inferior alveolar nerve following sagittal split ramus osteotomy in monkeys: a comparison of monocortical and bicortical fixation. Br J Oral Maxillofac Surg.

[45] Thygesen TH, Bardow A, Norholt SE, Jensen J, Svensson P (2009). Surgical risk factors and maxillary nerve function after Le Fort I osteotomy. J Oral Maxillofac Surg.

[46] Al-Nawas B, Wriedt S, Reinhard J, Keilmann A, Wehrbein H, Wagner W (2013). Influence of patient age and experience of the surgeon on early complications after surgical closure of the cleft palate--a retrospective cohort study. J Craniomaxillofac Surg.

[47] Cunningham SJ, Hunt NP, Feinmann C (1995). Psychological aspects of orthognathic surgery: a review of the literature. Int J Adult Orthodon Orthognath Surg.

[48] Frost V, Peterson G (1991). Psychological aspects of orthognathic surgery: how people respond to facial change. Oral Surg Oral Med Oral Pathol.

[49] Baherimoghaddam T, Tabrizi R, Naseri N, Pouzesh A, Oshagh M, Torkan S (2016). Assessment of the changes in quality of life of patients with class II and III deformities during and after orthodontic-surgical treatment. Int J Oral Maxillofac Surg.

[50] Miguel JA, Palomares NB, Feu D (2014). Life-quality of orthognathic surgery patients: the search for an integral diagnosis. Dental Press J Orthod.

[51] Choi WS, Lee S, McGrath C, Samman N (2010). Change in quality of life after combined orthodontic-surgical treatment of dentofacial deformities. Oral Surg Oral Med Oral Pathol Oral Radiol Endod.

[52] Soh CL, Narayanan V (2013). Quality of life assessment in patients with dentofacial deformity undergoing orthognathic surgery--a systematic review. Int J Oral Maxillofac Surg.

[53] Rustemeyer J, Gregersen J (2012). Quality of Life in orthognathic surgery patients: post-surgical improvements in aesthetics and self-confidence. J Craniomaxillofac Surg.

[54] Murphy C, Kearns G, Sleeman D, Cronin M, Allen PF (2011). The clinical relevance of orthognathic surgery on quality of life. Int J Oral Maxillofac Surg.

[55] Kavin T, Jagadesan AG, Venkataraman SS (2012). Changes in quality of life and impact on patients’ perception of esthetics after orthognathic surgery. J Pharm Bioallied Sci.

[56] De Araujo CM, Schroder AGD, De Araujo BMM, Cavalcante-Leao BL, Stechman-Neto J, Zeigelboim BS, Santos RS, Guariza-Filho O (2019). Impact of orthodontic-surgical treatment on quality of life: a meta-analysis. Eur J Orthod..

[57] Jung MH (2016). Quality of life and self-esteem of female orthognathic surgery patients. J Oral Maxillofac Surg.

[58] Burden DJ, Hunt O, Johnston CD, Stevenson M, O’Neill C, Hepper P (2010). Psychological status of patients referred for orthognathic correction of skeletal II and III discrepancies. Angle Orthod.

[59] Nguyen J, Duong H (2019) Anatomy, head and neck, mental nerve. In: StatPearls. Treasure Island (FL) 31536237

